# Concerns and priorities of Aboriginal and Torres Strait Islander peoples regarding food and nutrition: a systematic review of qualitative evidence

**DOI:** 10.1186/s12939-021-01551-x

**Published:** 2021-10-07

**Authors:** Rebecca Christidis, Mark Lock, Troy Walker, Mikaela Egan, Jennifer Browne

**Affiliations:** 1 Global Obesity Centre (GLOBE), Institute for Health Transformation, Deakin University, Locked Bag 20000, Geelong, Victoria Australia; 2 Victorian Aboriginal Community Controlled Health Organisation, 17-23 Sackville St, Collingwood, Victoria Australia

**Keywords:** Aboriginal and Torres Strait Islander, Indigenous, Food, Nutrition, Policy, Ecological model, Cultural safety, Qualitative research

## Abstract

**Background:**

Aboriginal and Torres Strait Islander Australians experience persistent health and social inequities. Chronic conditions, many of which are diet-related, are leading contributors to the burden of disease and health inequity in Australia. First Nations Peoples have the right to be involved in all policy decisions affecting them. This review aimed to synthesise Aboriginal and Torres Strait Islander Peoples’ concerns and priorities about food and nutrition in order to inform policies to improve health equity.

**Methods:**

MEDLINE, CINAHL, Informit and Google Scholar were systematically searched to identify qualitative studies–published from January 2008–that included data from Aboriginal and/or Torres Strait Islander Peoples about their concerns and priorities related to food and nutrition. Data were extracted from included studies using a pre-determined template and study quality was assessed using the Aboriginal and Torres Strait Islander Quality Appraisal Tool. Qualitative findings were synthesised using inductive thematic analysis and categorised based on an ecological model of health.

**Results:**

Twenty-one studies were included. Key factors influencing food and nutrition were identified across all levels of the ecological framework. These included interpersonal and institutional racism, junk food availability and marketing, food accessibility and affordability, housing conditions, food knowledge and cooking skills, and connection to family and culture.

**Conclusions:**

Documenting Aboriginal and Torres Strait Islander Peoples’ lived experiences of the colonised food system is one step necessary for informing policy to tackle food and nutrition inequities. Based on existing qualitative research, food and nutrition policymakers should prioritise building a supportive food environment by focusing on self-determination; ensuring access to healthy, affordable food and safe housing; and by eliminating systemic racism.

**Supplementary Information:**

The online version contains supplementary material available at 10.1186/s12939-021-01551-x.

## Background

Aboriginal and Torres Strait Islander Peoples, the First Nations Peoples of Australia, are recognised as one of the oldest continuous populations in the world [1]. Before colonisation, there were over 250 distinct language groups who lived in and cared for the lands now known as Australia for over 60,000 years [1, 2]. During this time, Aboriginal and Torres Strait Islander Peoples developed sustainable food systems instilled in cultural practices, intergenerational knowledge of seasonal food sources, and innovative food procurement and preparation techniques [3, 4]. For example, the Budj Bim Cultural Landscape, on the lands of the Gunditimara People in Western Victoria, is one of the world’s oldest and most complex aquaculture systems and is a UNESCO World Heritage site [5]. The waterways were deliberately constructed over six thousand years ago by the Gunditimara People, using volcanic rock to create channels, weirs, and dams to harvest eels and other food sources. Such examples of sustainable food systems and cultural ingenuity, passed down through generations provided a highly nutritious diet, free from refined fats, starch, sugar and salt, and protected First Nations People from many of the chronic diseases which are prevalent in Australia today [6].

The global subjugation of First Nations Peoples has resulted in a nutrition transition [7]. Following the invasion and subsequent colonisation of Australia, many Aboriginal and Torres Strait Islander Peoples were denied access to their traditional lands, cultural knowledge and food systems, through dispossession and forced assimilation [3, 8]. Traditional foods, high in protein, fibre and micronutrients were replaced—first, by government controlled rations and then to an imposed Western dietary pattern (high in sugar, starch, fatty meat and salt) which has resulted in a high prevalence of diet-related chronic disease [8, 9]. Like many First Nations Peoples worldwide, Aboriginal and Torres Strait Islander Peoples now experience significant social and health inequities, manifesting in a ten-year life expectancy gap with other Australians [10].

Health inequities are created by the conditions and systems in which people are born, live and age; which are, in turn, driven by social, economic and political factors [11]. For Aboriginal and Torres Strait Islander Peoples, over one-third of the health gap is explained by social determinants of health. This includes employment, income, education, housing, geography and ongoing colonisation and racism [12, 13]. These complex factors overlap with one another and with conventional ‘risk factors’ (including diet and obesity) to amplify health disparity [12]. Conversely, there is evidence demonstrating the protective effect of connection to culture, identity, family, community and ‘Country’ (a concept encompassing the interconnections between the physical elements, spirituality, identity, and culture) [14, 15].

Food and nutrition play an integral role in the physical, social and cultural wellbeing of Aboriginal and Torres Strait Islander peoples [16]. Much of the total burden of disease in Australian is due to chronic diseases, many of which are diet-related [17], and 15% of the health gap between Aboriginal and Torres Strait Islander and other Australians is attributable to dietary factors [17]. The rate of type 2 diabetes among Aboriginal and Torres Strait Islander adults is 3.3 times higher than for non-Indigenous adults, and one in four (23%) Indigenous households experience food insecurity [10]. Food insecurity is associated with economic disadvantage, insecure housing, environmental degradation and lack of access to traditional food systems [18, 19]. Fresh produce is often less accessible in remote areas, where Aboriginal and Torres Strait Islander Peoples represent 45% of the total population [10], and healthy food can cost up to 50% more in remote stores compared to capital cities [20]. Moreover, the prevalence of chronic conditions–such as type 2 diabetes and cardiovascular disease–is higher among Aboriginal and Torres Strait Islander Peoples living in remote and very remote areas, compared to those in major cities [21]. It follows that health inequities increase with remoteness [12]. The integral role of food and nutrition to overall wellbeing, in urban, regional, and remote Australia, has also been undermined by poor policy implementation.

Numerous policy responses have attempted to improve food and nutrition for Aboriginal and Torres Strait Islander Peoples. The first comprehensive food and nutrition policy, the *National Aboriginal and Torres Strait Islander Nutrition Strategy and Action Plan 2001-2010,* was developed through extensive consultation with Aboriginal and Torres Strait Islander communities [22]. It was not renewed following its expiry in 2010.

In 2007, the Council of Australian Governments (consisting of the chief ministers of Australia’s six states and two territories) developed joint agreements and set targets to improve the health and social equity for Aboriginal and Torres Strait Islander Peoples [23]. The targets included reducing the gaps in life expectancy, child mortality, education and employment between Aboriginal and Torres Strait Islander and other Australians [12]. The resulting ‘Closing the Gap’ strategy has achieved mixed results. Although there has been a 10% improvement in age-standardised mortality rates for Aboriginal and Torres Strait Islander People, this rate is like that of other Australians, thus the gap remains [12]. Food and nutrition were almost completely absent from the Closing the Gap agenda [24]. Furthermore, the policy framework was criticised for being government-driven and employing a deficits-based approach, focussing on overcoming disadvantage based on a non-Indigenous ‘ideal’, rather than recognising the many strengths and assets of Aboriginal and Torres Strait Islander communities [25]. Health equity advocates now emphasise for future targets, policies and strategies to be co-designed with Aboriginal and Torres Strait Islander stakeholders [23].

A more collaborative approach to policy formulation was established during the development of the current (2013-2023) *National Aboriginal and Torres Strait Islander Health Plan* [26] which recognises the centrality of culture to wellbeing; the need to address systemic racism; and the holistic definition of Aboriginal health that incorporates “the social, emotional and cultural well-being of the whole Community in which each individual is able to achieve their full potential as a human being” ([27] p. x). The Health Plan included a renewed focus on food security and nutrition; however, most of the recommended strategies remain unimplemented. Because of this, Aboriginal and Torres Strait Islander organisations continue to advocate for meaningful engagement in policy development and implementation to ensure that decisions are culturally safe and based on self-determination [28].

At the international level, the United Nations General Assembly adopted the *Declaration on the Rights of Indigenous peoples (UNDRIP)* in 2007 [29]. The declaration enshrines Indigenous peoples’ right to self-determination, affirming that “Indigenous Peoples have the right to participate in decision-making in matters which would affect their rights” (Article 18) and that governments should “consult and cooperate in good faith with the indigenous [sic] peoples concerned…before adopting and implementing legislative or administrative measures that may affect them” (Article 19) ([29] p. 18). Although Australia originally voted against the UNDRIP, it announced its support for it in 2009, and thus has the moral responsibility to implement it [30].

Aboriginal and Torres Strait Islander Peoples’ advocacy for self-determination in Australian policy development was exemplified by the release of the Uluru Statement from the Heart in 2017. This calls for a constitutionally enshrined voice to the Australian Parliament [31]. Rejected by the Australian government, this proposal provides a trajectory for self-determination of Aboriginal and Torres Strait Islander Peoples in line with UNDRIP.

In the field of First Nations Peoples’ food and nutrition, systematic reviews have focussed on quantitative evidence in order to identify which food policy actions are likely to be most effective [32–34]. They have identified a need to identify food and nutrition strategies which better align with diverse local knowledges [35]. The perspectives and priorities of Aboriginal and Torres Strait Islander Peoples concerning food and nutrition policy have not been systematically synthesised. In order to formulate equitable public policy which supports self-determination, it is essential that Aboriginal and Torres Strait Islander voices are included in the evidence review process. Therefore, with our team of First Nations and non-First Nations researchers, the review will seek to answer the following questions: What are the key factors influencing food security and nutrition, and which policy actions should be prioritised to improve food security and nutrition, with Aboriginal and Torres Strait Islander Peoples?

## Methods

We followed the Preferred Reporting Items for Systematic Reviews and Meta-Analyses guidelines [36] and the protocol was registered with PROSPERO (no: CRD42021226775). Our review team comprised three Aboriginal (ML, TW, ME) and two non-Aboriginal Australians (RC, JB) with qualifications and experience in public health, health promotion and/or nutrition. The review was part of a larger research project designed in partnership with the Victorian Aboriginal Community Controlled Health Organisation (VACCHO). Findings have been presented to VACCHO staff in order to inform future research, policy, and advocacy.

### Search strategy

A systematic literature search was undertaken across six electronic databases. Peer reviewed literature was identified using title and abstract searches in the MEDLINE, CINAHL (via EBSCOHost) and Informit (Health and Indigenous Collections) academic databases, and Google Scholar (first 100 hits), the *Australian Indigenous HealthInfoNet* and the *Australian Indigenous Health Bulletin* were used to search for grey literature. Date ranges were limited on all searches from 1^st^ January 2008 to November 2020, a timeframe that allowed for identification of contemporary literature published since the 2009 adoption of the UNDRIP. Reference lists of included studies were scanned for additional sources.

We used four sets of search terms based on the following categories: 1) Australian Aboriginal and Torres Strait Islander populations, 2) food and nutrition, 3) concerns and priorities, and 4) qualitative study designs. Search terms were entered one by one within the title (TI) and abstract (AB) fields of academic databases. Relevant subject headings were used in database searches when possible. Terms and subject headings within each category were combined with the Boolean operator ‘OR’. Finally, the four sets of terms were combined with the operator ‘AND’. The MEDLINE search strategy is available in Additional file 1. A similar strategy was used for the other databases.

### Study screening and selection

Identified studies were uploaded to the Covidence systematic review web application [37]. After duplicates were removed both initial title/abstract screening and full text screening was undertaken independently by two reviewers (RC and JB). Disagreements regarding study inclusion were resolved through discussion until consensus was reached. Articles were included if they were published in English after 1^st^ January 2008 and met the following criteria:

1. Included data from Australian Aboriginal and/or Torres Strait Islander participants. Studies with mixed populations were included if Indigenous status was identified in reporting of results.
2. Focused on healthy eating or population food and nutrition issues, including food security and dietary aspects of obesity/chronic disease prevention. Studies focussed on breastfeeding and specific therapeutic diets were excluded.
3. Reported the perspectives of Aboriginal and/or Torres Strait Islander People regarding factors influencing diet and/or priority actions to improve nutrition. Studies where only non-Aboriginal participants (e.g., health professionals) reported findings on behalf of Aboriginal/Torres Strait Islander peoples were excluded.
4. Original research using a qualitative research design (including the qualitative component of mixed methods studies). Reviews, commentaries, and protocols were excluded.

### Quality assessment

The quality of included studies was assessed using the Aboriginal and Torres Strait Islander Quality Appraisal Tool [38]. This tool was specifically designed for appraising research in the Australian Aboriginal and Torres Strait Islander context and comprises 14 questions that assess the quality of the research governance, community engagement, respect for cultural and intellectual property, and capacity building from an Aboriginal and Torres Strait Islander perspective. Two Aboriginal reviewers (TW and ML) independently appraised each of the included studies and disagreements were resolved through discussion with a third researcher (JB). Studies were considered high quality if they provided evidence for at least 10 of the 14 appraisal questions, moderate quality if 6–9 of the questions were satisfied, and low quality if 5 or less of the appraisal questions could be endorsed with reference to explicit statements in the text.

### Data extraction and analysis

A data extraction template was developed to compare the key characteristics of included studies: the study setting, study design, data collection methods, sample size, participant demographics and key findings. Data were extracted independently from all studies by two reviewers (RC and ML), one of whom was Aboriginal. Results were cross-checked by a third researcher (JB).

Studies were synthesised using qualitative thematic analysis [39]. Included studies, uploaded into NVIVO 12 software (QSR International), were inductively coded. Initial coding was undertaken independently by two reviewers (RC and JB) and were discussed in order to agree on a final coding framework. Descriptive themes were developed by grouping codes into similar concepts. Abstraction of findings into higher order interpretive themes, using the conversational language of study participants, was first undertaken by the same two reviewers, then discussed with all members of the research team until consensus was reached.

**Fig. 1:**
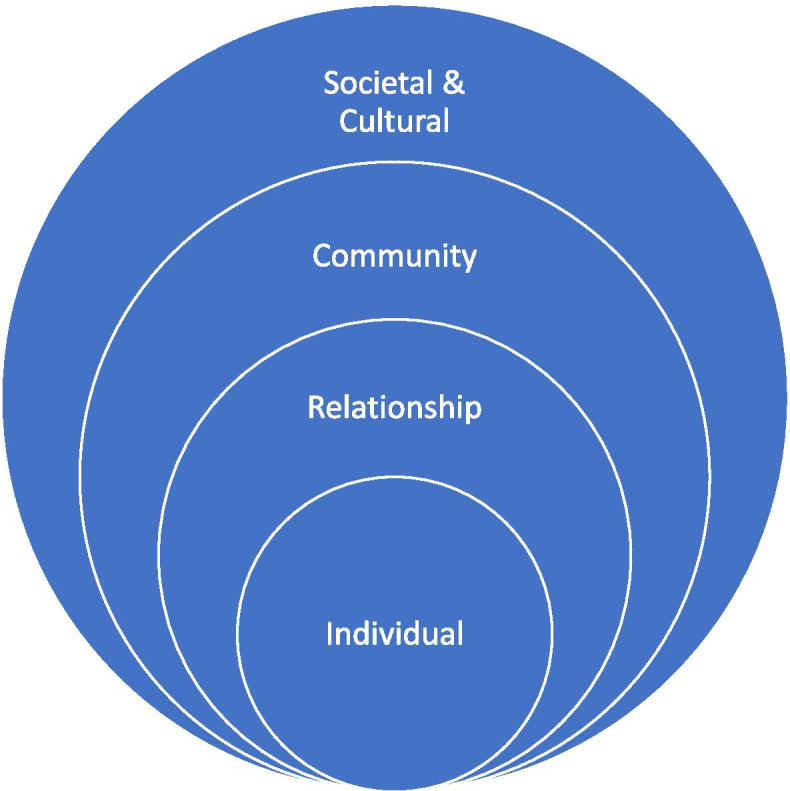
Ecological Framework. Reproduced with permission from Snijdjer et al. 2019

Themes identified in the literature were mapped against an ecological framework for understanding the determinants of health. Socioecological models recognise that health behaviours are influenced by dynamic factors in an individual’s immediate living, working and macro-socioeconomic environment [40]. Social-ecological theory is widely used in public health as it enables a holistic perspective of the determinants of health and the identification of public policies to reduce health inequity [41]. It depicts a systems thinking approach and is valuable to nutrition promotion for Aboriginal and Torres Strait Islander Peoples as it highlights the social, political, cultural, ecological and economic inequities underscoring the lived experience of food and diet [42]. We structured our findings according to the individual, relationship, community, societal and cultural levels of an ecological framework for Indigenous wellbeing proposed by Burnette et al [43], and further developed by Snijder et al. (Fig. 1) [44]. Similar frameworks have been used in other systematic reviews in the Aboriginal and Torres Strait Islander context [45–47].

## Results

**Fig. 2:**
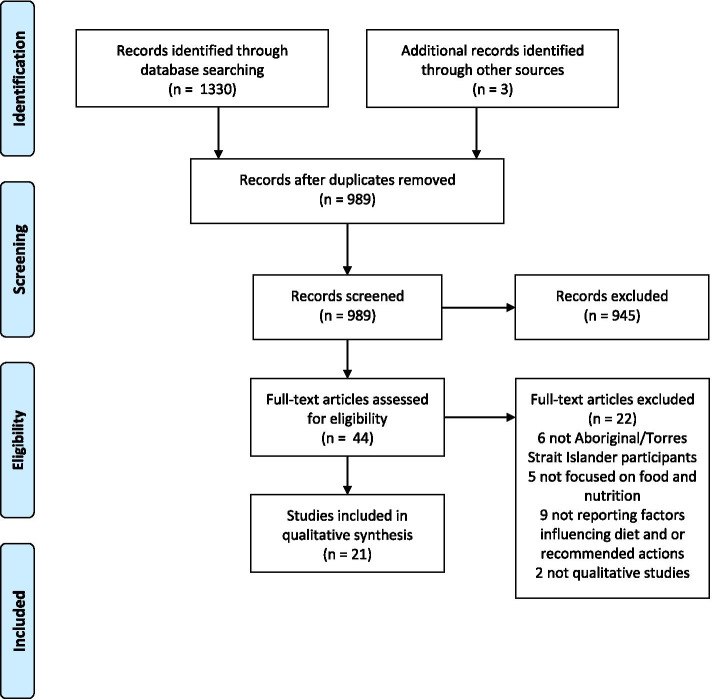
PRISMA Flow diagram of included and excluded studies (see Additional file 3 for complete PRISMA checklist)

The search strategy identified 985 studies, after duplicates were removed. There were forty-four full text studies assessed for eligibility, of which 18 journal articles and one doctoral thesis chapter met the selection criteria. A further three grey literature references were identified from Aboriginal Community-controlled health organisations, one of which was the full report of one of the included journal articles [48, 49]. Thus, this review included 22 references which reported 21 individual studies (Fig. 2). Included studies were published between 2009 and 2020 and all Australian jurisdictions were represented, except for Tasmania and the Australian Capital Territory. The Australian Statistical Geographical Standard (ASGS) Remoteness Structure was used to classify study locations [50]. There were six studies based in major cities, two in regional areas, eight in remote or very remote areas and five studies were undertaken across multiple urban/rural/remote locations.

The studies used a range of qualitative designs and data collection methods, including ethnography, focus groups, interviews, storyboarding and photo-voice and yarning sessions. Over 2,000 Aboriginal and/or Torres Strait Islander People participated in the studies. Most studies (*n*=17) involved participants aged 18-60 years. However, some studies also included children, teenagers, and Elders. See Table 1 for characteristics of included studies.

**Table 1:** Summary of included studies

Author/s	Location	Study design (Quality)	Total no. of Participants	Indigenous Participants n%	Age groups (years)	Key findings
Abbott et al. 2010 [51]	Urban, New South Wales	Qualitative evaluation, in-depth interviews (Moderate)	23 (19 female; 4 male)	100%	19-72	Key influences on diet included family; social and cultural importance of communal eating; cost of food; personal preference.
Adams et al. 2012 [52]	Urban, Victoria	Action research, photovoice (High)	10 (9 female; 1 male)	100%	Majority 20-39	Key factors influencing food choice included collectivism, creation of family harmony; concerns for hungry children, low vegetable intake. Aboriginal co-operative policy regarding healthy food provision at events
Brimblecombe et al. 2014 [53]	Remote, Northern Territory	Ethnographic- Semi-structured interviews, group discussions (Moderate)	46 (interviews with 16: 8 female; 8 male)	100%	35-60	Factors influencing food choice include transition from traditional to Western foods, lack of confidence in Western food system; food insecurity- low incomes, high food/living costs; reliance on cheap, energy-dense foods; inadequate food storage/refrigeration facilitates.
Bryce et al. 2020 [54]	Remote, South Australia	Indigenist and ethnographic: observations, conversation and reflections. (High)	14	100%	Unstated	Key factors influencing diet include high food prices; low income; overcrowded housing; availability of food storage/preparation/cooking facilities/equipment; reliance on take-away food; sharing resources enabled resilience
Colles et al. 2014 [55]	Remote, Northern Territory	Qualitative semi-structured interviews (Low)	30 (21 female; 9 male)	100%	18-60+	Inadequate supply of quality food, poor household infrastructure. Freedom of choice valued. Desire for practical food literacy/cooking skills. Preference for inter-personal education rather than pamphlets/flipcharts.
Cubillo et al. 2020 [56]	Urban, Northern Territory	Hermeneutic phenomenological approach, semi-structured interviews (Moderate)	12 (8 female; 4 male)	100%	18+	Traditional foods important for health and wellbeing. Concerns for loss of culture among younger generations. Access, availability, mobility, and local knowledge, social support networks influence traditional food consumption.
Cuesta-Briand et al. 2011 [57]	Urban, Western Australia	Qualitative descriptive study, focus groups, in-depth interviews (Low)	38 (28 female; 10 male)	47% (*n* = 18)	25-75+	Dependence on relatives for food provision/meal preparation. Food insecurity- high cost of food, low income, healthier food considered more expensive
Ferguson et al. 2017 [9]	Remote, Northern Territory and Western Australia	Mixed methods: interviews, observation, document review (Low)	54 (28 female; 26 male)	78%	<35(*n* = 6); >35(*n* = 48)	Strong support for food pricing policy to improve affordability of fruit and vegetables. Promotion and magnitude of discount key to effectiveness
Foley 2010 [58]	Urban, Queensland	Ethnographic: in-depth interviews, participant observation, group discussion. (Low)	31 (24 female; 7 male)	100%	18+	Key concerns included food affordability, lack of cooking ideas, keeping family members satisfied, access to nutrition information, extra work involved in healthy cooking. Cooking workshops provided opportunities to experiment with new recipes in a relaxed, social environment.
Lawrence 2015 [59]	Urban, New South Wales	Grounded theory: focus groups (Low)	40 (30 female; 10 male)	100%	16-66	Factors influencing food choice included nutrition knowledge; access to quality food; family perceptions of appropriate food and body weight, stress/depression. Supermarkets stocked with processed foods. Traditional food associated with food, identity, culture, wellbeing.
Lowell et al. 2018 [60]	Remote, Northern Territory	Ethnography: in-depth interviews, participant observation. (High)	36	100%	2 mo-2(*n* = 6); 18-70(*n* = 30)	Key challenges included housing insecurity/overcrowding; food insecurity: running out of money/going without food; reliance on takeaway food when unable to store food at home or unable to share with household.
McCarthy et al. 2018 [61]	Urban, Northern Territory	Qualitative inductive approach: initial interviews followed by in-depth interviews (Low)	30 (27 female; 3 male)	100%	17-58	Food security issues: food affordability, low income, high cost of living, limited supermarket access, inadequate housing infrastructure, social problems (e.g. gambling, alcohol). Family support provides safety net.
Mellor et al. 2016 [62]	Remote, rural and urban Western Australia and Victoria	Participatory action research: Semi-structured interviews, focus groups (Moderate)	150 (male)	100%	18-35	Barriers to healthy eating included disconnection from land, culture and traditional foods; food cost, nutrition knowledge/cooking skills; availability of fast food, discrimination/racism, lack of Aboriginal-specific services.
Murtha 2012 [63]	Remote, Queensland	Policy development consultation (Moderate)	Unstated	Unstated	Unstated	Four key priorities: 1. Healthy and strong Aboriginal and Torres Strait Islander People. 2. Adequate food supply, availability and access. 3. Keep track of nutrition indicators. 4. Build nutrition capacity.
Myers et al. 2014 [48]; VACCHO 2012 [49]	Urban and rural Victoria	Qualitative needs assessment: focus groups (Moderate)	35 (22 female; 13 male)	100%	18 +	Key issues included fussy easting in children, demand for sweet drinks,/junk foods; poor oral health, overweight, access to information.
Nilson et al, 2015 [64]	Rural, Western Australia	Ethnographic: participant observation, yarning groups, individual yarning sessions. (High)	17 (female)	100%	18-60	Key factors influencing nutrition included loss of traditional foods/cultural knowledge; shame caused by social inequity/racism; junk food advertising, cost of healthy food, easy access to fast food, family pressure.
Seear et al. 2020 [65]	Remote, Western Australia	Community directed participatory action research: focus groups (High)	32 (24 female; 8 male)	100%	16-17 (*n* = 15); 18-25 (*n* = 4); 45 (*n* = 13)	Key issues included availability/marketing of unhealthy food and sugary drinks; high cost/poor quality of fresh food in remote stores; inaccurate nutrition information.
Street et al. 2018 [66]	Remote, South Australia	Community deliberative forum, co-design using storyboard (Moderate)	14 (7 female; 7 male)	100%	18-34 (33%); 35-54 (33%); 55+ (33%)	Key issues included food cost and racism. Priority actions: teaching Aboriginal history in school; parenting classes; youth programs, improve school food environment, improve food literacy, cooking skills, food labelling
Thorpe and Browne 2009 [67]	Urban and rural, Victoria	Policy development consultation (High)	Unstated (consultations in 27 sites)	Unstated	Unstated	Action areas included: Increase Aboriginal nutrition workforce; access to affordable, nutritious and culturally appropriate food; policies to support healthy eating in key settings; funding for community nutrition programs; culturally appropriate health information/social marketing
Thurber et al. 2016 [68]	Remote and urban (11 sites around Australia)	Mixed methods: cross-sectional surveys/interviews, focus group (Low)	1230 (carer interviews) 12 (focus group)	100%	Unstated	Barriers to fruit and vegetable intake included access, financial status, resource status, housing, crowding and infrastructure, availability of produce in rural/remote area, children’s dislike, wasting money on uneaten food
Waterworth et al. 2016 [69]	Urban and rural, Western Australia	Participatory action research and constructivist grounded theory: focus groups (Moderate)	120 Urban: 70% female, Rural: 40% female,	100%	15->54 years	Key factors included living between two cultures; impact of colonisation; protective effect of culture; low income; inadequate housing; racism; mistrust of government/non-Indigenous programs/services). Personal choice important.

According to the Aboriginal and Torres Strait Islander Quality Appraisal Tool, six studies were rated high quality, eight were considered moderate quality, and seven were assessed as low quality. Most studies provided some evidence of community consultation and engagement. There were some studies that demonstrated Aboriginal and/or Torres Strait Islander leadership, governance and capacity building throughout the research process. There was no clear difference between academic and grey literature in terms of quality assessment. The quality assessment elements most frequently lacking were the protection of intellectual and culturally property rights; Aboriginal and Torres Strait Islander control over data collection and management; and plans to translate findings into sustainable changes in policy or practice (see Additional file 2).

### Nutrition concerns and priorities

There were nine themes for Aboriginal and Torres Strait Islander Peoples’ concerns and priorities regarding food and nutrition (Table 2). The themes are described in detail below and organised to align with the levels of the ecological framework [44]. Quotations from study participants are used to illustrate lived experiences.

**Table 2:** Summary of key themes identified in included studies

Ecological level	Themes
Cultural	Culture is central to health and wellbeing
Societal	The anguish and shame of racism
	It’s so easy to get junk food
	Accessing quality, fresh food can be hard
Community	It’s too expensive to eat healthy
	Good housing for good food
	Aboriginal organisations leading the way
Relationships	Food for the whole family
Individual	Learning to cook simple, healthy meals

#### Cultural-level factors

##### Theme 1: Culture is central to health and wellbeing

Connection to culture, particularly via traditional foods, was reported to be associated with positive health and wellbeing. Several studies reported that participants were enthusiastic about opportunities to discuss their traditional food experiences [52, 53, 56, 62, 69]. Conversely, disconnection from the traditional food systems was reported as a barrier to healthy eating and psychosocial wellbeing, as participants reported a sense of loss and imbalance when away from their homelands [52, 53, 56, 62, 69]. Some participants expressed concern that cultural knowledge was not being passed down to younger generations and could be lost. The role of culture in guiding children’s food choices was also demonstrated through storytelling to pass down knowledge from Elders to children. As highlighted by a mother:

*“By telling the story which is the healthy one and which is the bad or sweet one ... I tell them, this one helps you grow, gives you energy and vitamin for the blood”* ([55] p. 369).

Participants associated traditional foods with their identity and culture as well as with healthy eating. “*Bush tucker*” was highlighted as a way of incorporating healthy foods into the diet, and promoting wellbeing, although access to traditional foods was often limited ([59] p. 59). Some participants reported they could access traditional foods via commercial shops. Discussion of traditional foods was more common among participants in remote areas; however, concern regarding access to traditional foods was shared in both remote and urban communities, as illustrated by this quote from a Melbourne-based community member:

*“Having the opportunities to grow our own traditional foods and being able to teach our children…it’s one of the ways of ensuring survival for our culture.”* ([67] p. 26)

#### Societal-level factors

##### Theme 2: The anguish and shame of racism

Racism was emphasised as influencing health in many studies. The consequences of both historical, institutional and interpersonal racism on health and nutrition were emphasised by participants in urban, regional and remote areas. Many participants spoke about the forced dietary transitions that occurred following colonisation, including provision of rations in the missionary era, and contemporary experiences of racism in the Western industrialised food system [62, 64, 66]. One participant experienced racism within a supermarket as she was accused of shoplifting, after paying for goods via a self-serve checkout [66]. Others reported feeling ostracised and being subject to racial discrimination through the use of the *Basics Card* that restricts the spending of Aboriginal recipients of social security payments in the Northern Territory. As highlighted by a participant in a regional community:

*“I have seen the anguish in people’s faces when they go to get something: “Oh we don’t accept the Basic card” and the stress we go through [many in the group agreeing] and the shame when you walk away”* ([66] p. 130).

The impacts of systemic and interpersonal racism and discrimination included shame and embarrassment, as well as lowering self-confidence and self-esteem [64]. Stress caused by racism was reported by some participants as a deterrent from accessing health services. The fear of discrimination from health staff was reported by a participant:

*“If you ask if there is somewhere to go to get information [on healthy eating], you feel like they look at you. “Why do you want to know that?” Shame; no good”* ([64] p. 3399).

Recommendations for combatting systemic discrimination included teaching Aboriginal and Torres Strait Islander history and culture in schools [66], and increasing the numbers of Aboriginal staff in the health system to improve cultural safety [48, 57, 65, 69]. Trust and communication in the delivery of nutrition advice was fundamental, as people preferred a personal approach, rather than mainstream health promotion resources, such as a pamphlets [58, 69]. Parents were less likely to seek services if they felt they were being lectured to or did not feel empowered [48]. The importance of effective, culturally safe, locally relevant communication was frequently highlighted ([65] p. 5). For example, Aboriginal staff to provide culturally appropriate nutrition messages:

*“Trained (Aboriginal) nutrition workers. That’d be awesome … really good.”*

*“With someone like you to help with nutrition, you can understand all them kinds of foods and you know what’s in it … different stuff like that triangle, food group stuff”* ([48] p. 373).

##### Theme 3: It’s so easy to get junk food

The availability and marketing of unhealthy foods and drinks in the contemporary food environment was reported by many participants to provoke unhealthy eating. They regularly purchased fast foods, such as takeaway chicken and chips or chain burger meals, as they were seen as more manageable and less time-consuming [51, 68]. Shared frustration surrounding the overexposure to energy dense foods and sugary drinks was evident in comments made by two participants in a remote community:

*“Just tell them to just stop bringing cool drink here”*

*“Just go to the shop and tell them [laughing] to put all the junk food away”* ([65] p. 4).

In addition to the availability of unhealthy foods and drinks, the confusing nutrition messages on food packaging and on television were discussed by participants as a barrier to healthy eating. Children’s nutrition was a concern for families, as frequent exposure to the advertising of junk foods was associated by participants with fussy eating habits and a taste for junk foods from an early age [48]. Furthermore, false and misleading nutrition information negatively influenced food choice, as one participant living in a regional community of Western Australia explained:

*“Sometimes you don’t know about if it’s [food] good or bad and you can’t tell when the TV ads say it’s good; you believe them”* ([64] p. 3399).

##### Theme 4: Accessing quality, fresh food can be hard

Access to quality fresh foods was a concern highlighted in multiple studies—particularly in remote areas. Participants described limited access to high-quality fruits and vegetables, and described what was available as “*poor quality of fresh produce*” ([68] p. 834). Words such as “rubbish” or “poison” were also used to describe the foods purchased in supermarkets ([55] p. 367, 57 p. 57), referring to both processed food items and fresh produce. Participants also discussed difficulty in accessing healthy foods, including traditional foods and fruits and vegetables, due to mobility or transport issues. Access to supermarkets was considered important for sourcing affordable food, however, some participants only had a convenience store or petrol station within walking distance, where a limited number of items were available [61]. A reported barrier was “*Issues with public transport*” ([68] p. 834) to accessing quality produce both in remote and urban areas, especially for single parents—*“hard to take the bus with a baby and a two year old to go shop”* ([61] p. 9). Access to a reliable car was considered an asset, as a 29-year-old mother of five illustrated:

*“We didn’t have a car before but have one now. Made it easier to get around and do the shopping”* ([61] p. 9).

Despite the accessibility barriers experienced by some participants, close connection of Aboriginal communities was reported to be an enabler to food access. Participants with transport or mobility issues were able to reach out to family and social networks and Aboriginal community organisations for assistance with shopping and occasionally sourcing traditional foods [56, 57].

#### Community-level factors

##### Theme 5: It’s too expensive to eat healthy

Food affordability was the most common barrier to healthy eating identified by participants. They reported being on restricted incomes, such as social security payments or pensions and almost all studies highlighted experiences of not having enough money to purchase healthy food [57, 61], especially after paying bills or during the ‘off-pay’ week, when *“there’s not enough money, full stop”* ([61] p. 7). There were several reports of strict budgeting and selecting foods based on what was on special and needing to “stretch meals” in order to feed extended family or visitors ([58] p. 270). To compensate for financial instability, participants reported reducing the food budget, opting for cheaper, less healthy alternatives [53, 57]. This common challenge is illustrated in the following quote from a young Aboriginal mother:

*“Sometimes we have to be tight [with money] when the big bills (electricity, car repayments) come in and choose less expensive foods to buy”* ([61] p. 6)

In general, healthier foods were perceived as more expensive across urban, regional and remote areas. Satiating meals took preference over selecting foods based on health or nutrition. For example, “bulking up” with low-cost starchy foods such as rice, pasta or white bread [52, 61]. Products lower in sugar or saturated fat and higher in fibre were reported to be more costly and not within the regular food budgets of some participants [57]. Food subsidies or price discounts to make healthy foods more affordable was a strategy supported by participants in several studies [63, 66, 70]. As one participant noted:

*“Healthy food’s always a bit dearer. Like, white bread’s a dollar, multigrain and wholemeal’s a dollar seventy-nine […] Why isn’t wholegrain and multigrain a dollar?”* ([49] p. 51)

##### Theme 6: Good housing for good food

Inadequate and insecure housing conditions, in remote and urban areas, were barriers to healthy eating across studies. There were multiple reports of inadequate facilities to safely prepare and store foods [61, 68]. Food wastage was also a concern as many participants reported not having access to appropriate refrigeration and storage facilities in the home [51]. McCarthy et al. documented participants’ experiences with poor housing conditions and landlord negligence, illustrated by this quote from an Aboriginal mother:

*“… We’ve told him [owner] about the kitchen cupboards falling apart and other problems in the house. Just doesn’t seem to want to do anything about it...”* ([61] p. 10)

Unstable living circumstances was associated with lack of control over of participants’ immediate environment, resulting in reduced nutritional and psychosocial wellbeing [53, 60, 62]. Psychosocial stressors caused by living in overcrowded houses contributed to consumption of convenience foods [51]. Participants explained that living with many other people made it difficult to cook and eat healthy meals, as they could not afford to share with everyone in the household. Instead, takeaway meals were often eaten outside of the home to avoid unachievable food requests from others [53, 60].

##### Theme 7: Aboriginal organisations leading the way

Aboriginal organisations (health services, childcare centres, and remote food stores) were named as key sites for nutrition promotion. They were involved in food provision [56, 57], cooking [51, 58, 64] and nutrition education programs [48, 55, 63]. The importance of programs and services–designed for and led by–Aboriginal and Torres Strait Islander people was emphasised in many studies [51, 62, 66, 69]. The importance of sustainable funding for community nutrition programs was also raised [67]. The need for Aboriginal organisations was explained by this participant:

“*If we had our own Aboriginal centre things would be different, ’cause we can bring all our stuff to that centre, cook our food, and learn our ways”* [69]

Another strength of Aboriginal organisations was the leadership role they played in providing healthy food environments. Food stores in remote areas, which are owned by the local Aboriginal community, have implemented a range of policies to improve the supply and affordability of healthy foods [70]. Some Aboriginal health services and childcare centres had nutrition policies in place so that healthy food was provided [48, 52]. In consultations led by Aboriginal community controlled organisations, participants expressed support for nutrition policies to ensure healthy food was provided at community events [49, 63, 67]. One study identified schools as an important setting for providing healthy food environments through healthy canteens [66].

#### Relationship-level factors

##### Theme 8: Food for the whole family

Family was identified as an important influence of food choice that could be both an enabler and barrier to healthy eating. Having family support and healthy role models, in both the immediate and extended family, was described as a key facilitator of healthy eating [51, 53, 66, 69]. The importance of individual freedom, including children’s autonomy regarding food choice, was often discussed [51, 55, 69]. Children’s liking (or dislike) for healthy food such as fruit and vegetables was a key factor influencing food choice [68], with some parents reporting reluctance about introducing healthy foods to their children for fear of rejection, and food wastage [52, 58]. Giving in to children’s demands for junk food was an experience shared by many participants, *“sugary foods – gives the mother peace”* ([48] p. 372). Some participants reported feelings of powerlessness when trying to influence the family diet. One Elder described her situation regarding the influence of family:

*“The kids bring take-aways. I just can’t say no, it’s horrible. I just can’t control it. […] I just couldn’t go on a diet…”* ([57] p. 386).

Due to the strong influence of family on food choice and eating patterns, many studies suggested that individual nutrition advice was unlikely to be effective and that, instead, nutrition promotion should be family focussed [49, 58, 64]. Similarly, parenting skills, cooking and nutrition programs for young families were a recommended strategy to increase confidence around providing healthy foods for children [66, 67].

#### Individual-level factors

##### Theme 9: Learning to cook simple, healthy meals

Many study participants demonstrated a knowledge of healthy eating to prevent chronic diseases. For example, participants reported a healthy diet meant drinking water; consuming fruits and vegetables; and cutting back on sugar and fat–as these foods were associated with “fat in the blood”, causing blockages “inside the chest” and “heart attack” ([55] p. 367, 61 p. 385). Participants also demonstrated an awareness of the association between diet and other health conditions, such as diabetes and oral health [51]. While some participants expressed a need for more nutrition education and information, particularly in schools [62, 66]; more common were requests for practical, hands-on programs to develop healthy shopping, recipe modification and cooking skills. Quick and easy-to-prepare recipes were reported as an enabler for healthy eating for that could potentially encourage children to try new foods and counter the temptation to buy takeaway [49, 51, 58]. As one mother reported:

*“I don’t have the time to cook tea so then there’s take-away ... yeah, I wanna eat healthy ... a real big issue for me is how do you prepare a nutritious meal, what is a nutritious meal?”* ([48] p. 372).

## Discussion

This is the first systematic review of qualitative literature describing Aboriginal and Torres Strait Islander Peoples’ concerns and priorities regarding food and nutrition. While previous reviews have provided evidence on the effectiveness of nutrition interventions [32–34], our focus on synthesising qualitative evidence privileges the voices of Aboriginal and Torres Strait Islander Peoples, in line with their right to self-determination [29].

We identified 21 studies, published over the past 12 years, representing the voices of over 2,000 Aboriginal and/or Torres Strait Islander people from across Australia. We identified key food and nutrition issues across each level of an ecological framework. At the macro (cultural and societal) levels, we found that culture, racism, food availability and junk food marketing were key determinants of nutritional health. At the meso (community) level we found that food affordability, housing conditions, transport, and Aboriginal organisations influenced eating patterns. At the micro (relationship and individual) levels, the family environment and food knowledge and skills were key drivers of food choice. It was also apparent in the articles that, while each theme appears discrete whereas, in reality, they affect each other, in line with the holistic view of health [15]. These findings can inform intervention points for improving nutrition at the different ecological levels, as well as the interaction of factors across levels.

These findings contribute to growing evidence that culture is central to Aboriginal and Torres Strait Islander Peoples’ health and wellbeing. Waterworth et al. (2016) show how culture is influential across the socioecological framework, and not just at the macro level [69]. Cultural determinants of health, including connection to Country, family, traditional knowledge, identity and cultural practices, are now recognised as having a protective effect on wellbeing [71]. In the current review, traditional foods were highly valued as way of connecting to culture as well as improving nutrition; however, access to them was often limited. International evidence suggests that traditional food knowledge and culture are closely linked with empowerment, self-determination and healthy eating [72]. Self-determination is a cultural determinant of health and is recognised as a human right for Indigenous Peoples [29]. Evidence from Native American communities demonstrates that when they make their own decisions about what actions to take, they consistently out-perform external non-Indigenous decision makers [73]. In the Australian context, this review highlighted the leading role Aboriginal organisations play, when adequately resourced, in providing culturally safe nutrition programs and healthy food environments.

An important finding of this review was that racism, both within the health system and the food retail sector, is a key barrier to improving nutrition. The link between racism and health inequities is now well-established [74–76]. For Aboriginal children and young people, racism is associated with poor mental health and cardiometabolic risk [77, 78]. More recently, a systematic review found an association between racism and obesity [79]. Eliminating racism in all sectors of society is critical to improving health equity and has been identified in the *National Aboriginal and Torres Strait Islander Health Plan* [26]. One strategy to address racism in the short term, identified in this review, is employing more Aboriginal and Torres Strait Islander health staff to deliver nutrition promotion programs [48, 57, 65, 69]. Western approaches to health and mainstream health promotion methodologies frequently overlook Aboriginal cultural knowledge and perspectives about food and nutrition [80, 81]. Although increasing the number of Aboriginal and Torres Strait Islander health professionals is important, other factors need to be addressed. These include management support, organisational culture, access to services, and other systemic factors that may improve cultural safety within the mainstream health system [82]. For example, a longer term strategy–suggested by participants in one study–was school programs to increase understanding of Aboriginal history and culture [66]. Awareness, among non-Indigenous health staff, of the inequities ascribed to colonisation and racism is an important step towards building culturally safe food and nutrition services [81, 82].

The availability and marketing of junk food is a key environmental driver of unhealthy eating [83]. Australian television has one of the highest frequencies of food advertising in the world and food marketing is dominated by unhealthy products [84]. Recent evidence suggests Australian children are also exposed to large amounts of junk food marketing online, via social media and in the built environment [85, 86]. The findings from this review indicate that Aboriginal and Torres Strait Islander people are concerned about the impact of unhealthy food and beverage marketing, particularly on children. Public health advocates have recommended the Australian Government implement stricter regulation of advertising for unhealthy foods and beverages [87, 88]. Evidence suggests that population-wide advertising restrictions are likely to be effective across socioeconomic groups [89]. However, the specific impact of restricting food advertising on First Nations Peoples has not been evaluated [32]. Additionally, evidence from Aotearoa/New Zealand suggests Māori children have higher rates of exposure to food marketing compared with non-Māori children [90]. If this is also the case in Australia, restricting junk food marketing, at least at the population level, may improve health equity for Aboriginal and Torres Strait Islander Peoples. Any government regulation should be balanced against self-determination rights to freedom of choice.

Aboriginal and Torres Strait Islander participants in studies included in this review have identified insecure and overcrowded housing with inadequate equipment and infrastructure for food preparation and storage, as a key barrier to good nutrition, consistent with previous research [19, 91]. In 2014-15, 29% of Aboriginal and Torres Strait Islander Australians were living in a house with major structural problems and one in five (19%) were living in a house that did not meet acceptable standards [92]. There is a need to improve household infrastructure, particularly in social housing, where one in seven (14%) residents are Aboriginal or Torres Strait Islander [92]. Mandating minimum standards in social housing to ensure adequate food preparation and storage facilities is a recommended priority.

A consistent finding across almost all studies included in this review, were the financial and physical access barriers to healthy eating [49, 51, 53, 56, 57, 59, 60, 62–65, 68, 69]. It has been reported that a diet consistent with the Australian Dietary Guidelines is largely unachievable for low-income earners [93], particularly among those who receive social security payments, for whom the cost of a healthy diet is reportedly 40% of disposable income [94]. This proportion can be as high as 80% in remote areas and at least one in five Aboriginal and Torres Strait Islander households report running out of food in the previous 12 months [10, 95]. One strategy for improving food security, identified by studies in this review, is healthy food subsidies*.* Previous research demonstrated subsidised fruit and vegetables cost increases fruit and vegetable purchasing, including in remote Aboriginal communities [96, 97]. To improve health equity, subsidies are recommended for improving the relative cost of fresh produce for low-income households [20].

Besides the broader food environment, this review found that food and nutrition knowledge and skills were also important factors influencing diets. Specifically, study participants frequently demonstrated a desire to increase their confidence in preparing healthy, simple, affordable family meals [55, 62, 64, 67]. Previous systematic reviews have found that practical, community directed nutrition education programs can be effective at improving dietary intake and health outcomes [32, 98]. However, it is important to note that a systems approach is required to improve food security and nutrition, and this cannot be achieved through education alone [99]. This review confirms that Aboriginal and Torres Strait Islander Peoples’ diets are influenced by a multitude of systemic and environmental factors: availability, marketing, affordability, physical access, and housing.

This review contributes evidence that can be used to inform equitable public health policy making in Australia. The Australian government is currently developing population wide strategies to address preventative health and obesity, as well as a new strategy for improving Aboriginal and Torres Strait Islander health equity. The synthesis of 21 relevant studies, representing the voices of diverse Aboriginal and Torres Strait Islander communities from urban, regional and remote locations across Australia, provides a rich source of intelligence for food and nutrition policy decision making. While this review does not replace the need for consultation, it makes an important contribution to the evidence base because the formal documentation of Aboriginal and Torres Strait Islander voices have been under-represented in national nutrition policy development processes [100]. Although a large number of Aboriginal and Torres Strait Islander Peoples participated in the studies within this review, it is important to note that their voices have been filtered through the mostly non-Indigenous researchers who authored the included studies.

Many of the themes related to food and nutrition identified in this review are not unique to Aboriginal and Torres Strait Islander Peoples. Culture, racism, family, food supply and self-determination are likely to be relevant to other Indigenous populations who have a shared history of colonisation, dispossession, marginalisation and disruption of cultural and kinship systems [32]. Moreover, issues related to food marketing, food affordability, housing, transport and food knowledge and skills, influence food choice for many individuals—especially those experiencing socioeconomic disadvantage [101]. Therefore, the priorities we have identified could contribute to improving health equity more broadly, both in Australia and in other high-income countries; however, contextually relevant implementation and evaluation is required to confirm this.

### Strengths and limitations

The strengths of this review include its systematic search strategy, broad eligibility criteria, and use of an established conceptual framework to organise the findings. Another strength is the inclusion of First Nations researchers on the review team and application of a quality assessment tool specifically designed for appraising Aboriginal and Torres Strait Islander health research [38]. Most studies within this review were rated low (*n* = 7) or moderate (*n* = 8) quality; however, it is important to note that the Aboriginal and Torres Strait Islander Quality Appraisal Tool was published in 2020 and the included studies dated back to 2009. As a result, we were looking backwards and assessing older studies with current quality standards. Additionally, it is possible that–in practice–some studies met more of the items on the appraisal checklist but this was not documented in the article due to journal word limits or the preferences of peer-reviewers or editors. For this reason, many studies scored ‘unclear’ in certain appraisal characteristics, thus our final assessment may have underestimated the quality of some studies. None of the themes or recommendations generated in this review were based on the findings of low-quality studies alone.

As with all systematic reviews, our synthesis may be limited by publication bias. We attempted to minimise this risk by including grey literature in our search strategy. We did not, however, include searches of government websites for consultation reports or policy submissions made by Aboriginal or Torres Strait Islander individuals or organisations related to food and nutrition. It is likely that such documents would also be a valuable source of data on this topic. This review is also limited by its lack of representation of Aboriginal and Torres Strait Islander young people and those living in the Australian Capital Territory and Tasmania. We also acknowledge the diversity of Aboriginal and Torres Strait Islander Peoples and do not assume that the voices included in this review represent the views or experiences of all First Nations Australians. Despite these limitations, we provide some high-level recommendations to inform policy and practice, keeping in mind the variable quality of the studies.

## Conclusion

Our findings suggest that action is needed at the macro-, meso- and micro-environmental levels. At the micro-environmental level, nutrition promotion professionals should work with families to improve practical food skills in a culturally safe and social environment—as determined by those families. At the meso-level, community housing, organisations, schools and food stores, are key venues for building healthy food environments to ensure access to healthy food and appropriate food storage/preparation facilities. At the macro-level, systemic change is needed to improve availability and affordability of healthy food (including traditional foods), and improve adequate housing, reduce the availability and marketing of junk food, and to eliminate racism. Culture and self-determination should be at the centre of all policy actions to improve food and nutrition with Aboriginal and Torres Strait Islander Peoples.

## Supplementary Information

**Additional file 1.** Search strategy.

**Additional file 2.**

**Additional file 3.** PRISMA 2020 Checklist.

## Data Availability

Not applicable. All included articles are publicly available
