# Supplementary material for: Concerns and priorities of Aboriginal and Torres Strait Islander peoples regarding food and nutrition: a systematic review of qualitative evidence
Source: Int J Equity Health. 2021 Oct 7;20:220. doi: 10.1186/s12939-021-01551-x (PMC8499519; doi:10.1186/s12939-021-01551-x)
Supplement: Supplementary file 1 — Additional file 1. Search strategy. [file 12939_2021_1551_MOESM1_ESM.docx]

**Additional file 1: Search strategy**

**Medline**

| TI OR AB | Aborigin* “Torres Strait Island*” Indigen* “First people*” First Nation*” “Remote N3 Communit*”  MH Oceanic Ancestry Group | Search with AND |
| --- | --- | --- |
| TI OR AB | Food* Diet* Nutrition* Eat* Fruit* Vegetable* Obes* Overweight* Feeding “Body Mass Index” BMI “Food insecure*” “Food secur*”  MH Diet, Food and Nutrition + |  |
| TI OR AB | Factor* Influence* Determin* Barrier* Enabl* Constrain* Obstacle* Difficult* Imped* Challeng* Hurdle* Obstruct* Facilitat* Perceive* Perception* belie* expect* strateg* perspective* reflect* concern* priorit* recommend* policy policies action* “community view*” “lived experience*”  MH Health Priorities |  |
| TI OR AB | Qualitative* Interview* “Focus Group*” ethnograph* consult* “needs assessment*” forum* yarn* story* stories gathering* “action research*” (participat* N2 research*)  MH Focus Groups  MH Qualitative Research  MH Community-based Participatory research |  |

*****indicates truncation

**CINAHL**

| TI OR AB | Aborigin* or "Torres Strait Island*" or Indigen* or “First people*” or First Nation*” or (Remote N3 Communit*) | Search with AND |
| --- | --- | --- |
| TI OR AB | Food* OR Diet* OR Nutrition* OR Eat* OR Fruit* OR Vegetable* OR Obes* OR Overweight* OR Feeding OR “Body Mass Index” OR BMI |  |
| TI OR AB | Factor* OR Influence* OR Determin* OR Barrier* OR Enabl* OR Constrain* OR Obstacle* OR Difficult* OR Imped* OR Challeng* OR Hurdle* OR Obstruct* OR Facilitat* OR Perceive* OR Perception* OR belie* OR expect* OR strateg* OR perspective* OR reflect* OR concern* OR priorit* OR recommend* OR policy OR policies OR action* OR "community view*" OR "lived experience*" |  |
| TI OR AB | Qualitative* OR Interview* OR “Focus Group*” OR enthnograph* OR consult* OR “needs assessment*” OR forum* OR yarn* OR story* OR stories* OR gathering* OR “Action research*” OR (participat* N2 research*) |  |

*****indicates truncation

|  |  |
| --- | --- |

**Informit**

| ((( TI:BMI* OR AB:BMI*) OR ( (TI:"Body Mass Index*") OR (AB:"Body Mass Index*")) OR ( TI:feeding* OR AB:feeding*) OR ( TI:overweight* OR AB:overweight*) OR ( TI:obes* OR AB:obes*) OR ( TI:vegetable* OR AB:vegetable*) OR ( TI:fruit* OR AB:fruit*) OR ( TI:eat* OR AB:eat*) OR ( TI:nutrition* OR AB:nutrition*) OR ( TI:diet* OR AB:diet*) OR ( TI:food* OR AB:food*)) AND (( (TI:"first nation*") OR (AB:"first nation*")) OR ( (TI:"First People*") OR (AB:"First People*")) OR ( (TI:"Torres Strait Island*") OR (AB:"Torres Strait Island*")) OR ( TI:Aborigin* OR AB:Aborigin*))) AND (( (TI:"Arnhem Land*") OR (AB:"Arnhem Land*")) OR ( (TI:"Cape York*") OR (AB:"Cape York*")) OR ( TI:Tasmania* OR AB:Tasmania*) OR ( (TI:"Northern Territor*") OR (AB:"Northern Territor*")) OR ( TI:Queensland* OR AB:Queensland*) OR ( (TI:"New South Wales*") OR (AB:"New South Wales*")) OR ( TI:Victoria* OR AB:Victoria*) OR ( TI:Australia* OR AB:Australia*)) AND (( (TI:"lived experience*") OR (AB:"lived experience*")) OR ( (TI:"community view*") OR (AB:"community view*")) OR ( TI:action* OR AB:action*) OR ( TI:policies OR AB:policies) OR ( TI:policy OR AB:policy) OR ( TI:recommend* OR AB:recommend*) OR ( TI:priorit* OR AB:priorit*) OR ( TI:concern* OR AB:concern*) OR ( TI:reflect* OR AB:reflect*) OR ( TI:perspective* OR AB:perspective*) OR ( TI:strateg* OR AB:strateg*) OR ( TI:expect* OR AB:expect*) OR ( TI:belie* OR AB:belie*) OR ( TI:perception* OR AB:perception*) OR ( TI:facilitat* OR AB:facilitat*) OR ( TI:obstruct* OR AB:obstruct*) OR ( TI:hurdle* OR AB:hurdle*) OR ( TI:challeng* OR AB:challeng*) OR ( TI:imped* OR AB:imped*) OR ( TI:difficult* OR AB:difficult*) OR ( TI:obstacle* OR AB:obstacle*) OR ( TI:constrain* OR AB:constrain*) OR ( TI:enabl* OR AB:enabl*) OR ( TI:barrier* OR AB:barrier*) OR ( TI:determin* OR AB:determin*) OR ( TI:influence* OR AB:influence*) OR ( TI:perceive* OR AB:perceive*) OR ( TI:factor* OR AB:factor*)) AND (( TI:BMI* OR AB:BMI*) OR ( (TI:"Body Mass Index*") OR (AB:"Body Mass Index*")) OR ( TI:feeding* OR AB:feeding*) OR ( TI:overweight* OR AB:overweight*) OR ( TI:obes* OR AB:obes*) OR ( TI:vegetable* OR AB:vegetable*) OR ( TI:fruit* OR AB:fruit*) OR ( TI:eat* OR AB:eat*) OR ( TI:nutrition* OR AB:nutrition*) OR ( TI:diet* OR AB:diet*) OR ( TI:food* OR AB:food*)) AND (( (TI:"first nation*") OR (AB:"first nation*")) OR ( (TI:"First People*") OR (AB:"First People*")) OR ( (TI:"Torres Strait Island*") OR (AB:"Torres Strait Island*")) OR ( TI:Aborigin* OR AB:Aborigin*)) |
| --- |

*indicates truncation

**Google Scholar**

Australia (Aboriginal OR "Torres Strait" OR Indigenous) (Food OR diet OR nutrition OR eating OR obesity) (Factor OR determinant OR barrier OR facilitator OR enabler OR perspective OR “community view”) Qualitative

**Healthinfonet**

| Title and abstract search | (diet, nutrition, overweight, obesity, food, eat, intake, fruit, vegetable) (priority, concern, barrier, facilitator, enabler, hurdle, perception, view, “community view”) (qualitative, story, yarn) |
| --- | --- |
