# Supplementary material for: Concerns and priorities of Aboriginal and Torres Strait Islander peoples regarding food and nutrition: a systematic review of qualitative evidence
Source: Int J Equity Health. 2021 Oct 7;20:220. doi: 10.1186/s12939-021-01551-x (PMC8499519; doi:10.1186/s12939-021-01551-x)
Supplement: Supplementary file 2 — Additional file 2. [file 12939_2021_1551_MOESM2_ESM.docx]

|  | Indigenous governance | | | | Respect for cultural and intellectual property | | | | Capacity building | | | Beneficial outcomes | | | Overall assessment |
| --- | --- | --- | --- | --- | --- | --- | --- | --- | --- | --- | --- | --- | --- | --- | --- |
|  | **Q1** | **Q2** | **Q3** | **Q4** | **Q5** | **Q6** | **Q7** | **Q8** | **Q9** | **Q10** | **Q11** | **Q12** | **Q13** | **Q14** |  |
| Abbott et al., 2010 | Y | Y | Y | Y | Y | U | U | P | U | P | N | P | Y | U | Moderate |
| Adams et al., 2012 | Y | Y | Y | Y | Y | U | P | P | Y | Y | Y | Y | Y | Y | High |
| Brimblecomb et al., 2014 | N | Y | Y | Y | Y | U | U | U | N | P | U | U | Y | P | Moderate |
| Bryce et al. 2020 | Y | Y | Y | Y | Y | U | U | Y | Y | P | Y | Y | P | P | High |
| Colles et al., 2011 | N | P | Y | U | U | U | U | U | U | N | U | U | Y | U | Low |
| Cubillo et al., 2020 | Y | P | Y | Y | P | U | U | U | Y | Y | U | U | P | P | Moderate |
| Cuesta-Briand et al., 2011 | U | U | U | U | U | U | U | U | U | U | U | U | U | U | Low |
| Ferguson et al., 2017 | P | P | U | Y | U | U | U | U | N | U | U | U | U | U | Low |
| Foley 2010 | N | Y | U | U | Y | U | U | U | N | U | U | P | U | P | Low |
| Lawrence 2015 | Y | P | Y | U | U | U | U | U | P | U | U | U | Y | U | Low |
| Lowell et al., 2018 | Y | Y | Y | Y | Y | U | P | U | P | Y | N | N | Y | Y | High |
| McCarthy et al., 2018 | Y | U | Y | U | U | U | U | U | U | U | U | U | Y | P | Low |
| Mellor et al., 2016*.* | N | P | N | N | U | U | U | P | N | P | N | U | P | Y | Moderate |
| Murtha 2012 | Y | P | Y | Y | P | U | U | U | P | U | Y | P | U | U | Moderate |
| Myers et al., 2014 | Y | Y | Y | Y | Y | U | U | U | U | P | U | P | Y | P | Moderate |
| Nilson et al., 2015 | Y | Y | Y | Y | Y | U | U | P | Y | Y | P | P | Y | Y | High |
| Seeara et al., 2020 | Y | Y | Y | Y | Y | U | U | P | P | P | P | Y | Y | Y | High |
| Street et al., 2018 | P | Y | U | Y | P | U | U | U | P | Y | U | P | Y | Y | Moderate |
| Thorpe & Browne 2009 | Y | Y | Y | Y | Y | U | U | Y | P | Y | Y | Y | P | U | High |
| Thurber et al., 2016 | P | U | N | Y | U | U | U | N | U | P | N | U | U | P | Low |
| Waterworth et al., 2015 | U | Y | U | Y | Y | U | U | U | P | Y | U | Y | P | Y | Moderate |

Y = yes, P = partially, U = unclear, N = no explicit statements in the body of the text to provide evidence for each question below

1. **Did the research respond to a need or priority determined by the community?**
2. **Was community consultation and engagement appropriately inclusive?**
3. **Did the research have Aboriginal and Torres Strait Islander research leadership?**
4. **Did the research have Aboriginal and Torres Strait Islander governance?**
5. **Were local community protocols respected and followed?**
6. **Did the researchers negotiate agreements in regards to rights of access to existing Aboriginal and Torres Strait Islander peoples’ intellectual and cultural property?**
7. **Did the researchers negotiate agreements to protect Aboriginal and Torres Strait Islander peoples' ownership of intellectual and cultural property created through the research?**
8. **Did Aboriginal and Torres Strait Islander peoples and communities have control over the collection and management of research materials?**
9. **Was the research guided by an Indigenous research paradigm?**
10. **Does the research take a strengths-based approach, acknowledging and moving beyond practices that have harmed Aboriginal and Torres Strait peoples in the past?**
11. **Did the researchers plan to and translate the findings into sustainable changes in policy and/or practice?**
12. **Did the research benefit the participants and Aboriginal and Torres Strait Islander communities?**
13. **Did the research demonstrate capacity strengthening for Aboriginal and Torres Strait Islander individuals?**
14. **Did everyone involved in the research have opportunities to learn from each other?**
